# DNA Methyltransferase Inhibitor Promotes Human CD4^+^CD25^h^FOXP3^+^ Regulatory T Lymphocyte Induction under Suboptimal TCR Stimulation

**DOI:** 10.3389/fimmu.2016.00488

**Published:** 2016-11-08

**Authors:** Chun-Hao Lu, Cheng-Jang Wu, Cheng-Chi Chan, Duc T. Nguyen, Kuo-Ray Lin, Syh-Jae Lin, Li-Chen Chen, Jeffrey Jong-Yong Yen, Ming-Ling Kuo

**Affiliations:** ^1^Department of Microbiology and Immunology, Graduate Institute of Biomedical Sciences, College of Medicine, Chang Gung University, Taoyuan, Taiwan; ^2^Division of Biological Sciences, University of California San Diego, La Jolla, CA, USA; ^3^Institute of Biomedical Sciences, Academia Sinica, Taipei, Taiwan; ^4^Department of Pediatrics, Division of Allergy, Asthma, and Rheumatology, Chang Gung Memorial Hospital, Taoyuan, Taiwan; ^5^Chang Gung Immunology Consortium, Chang Gung Memorial Hospital, Chang Gung University, Taoyuan, Taiwan

**Keywords:** immunological tolerance, human regulatory T cell, FOXP3, suboptimal TCR stimulation, epigenetic regulation

## Abstract

The “master transcription factor” FOXP3 regulates the differentiation, homeostasis, and suppressor function of CD4^+^ regulatory T (Treg) cells, which are critical in maintaining immune tolerance. Epigenetic regulation of FOXP3 expression has been demonstrated to be important to Treg cell development, but the induction of human Treg cells through epigenetic modification has not been clearly described. We report that the combination of the DNA methyltransferase inhibitor 5-azacytidine (5-Aza) and suboptimal T cell receptor (TCR) stimulation promoted CD4^+^CD25^h^FOXP3^+^ T cell induction from human CD4^+^CD25^−^ T cells. 5-Aza treatment enhanced the expression of Treg cell signature genes, such as CD25, FOXP3, CTLA-4, and GITR, in CD4^+^CD25^h^ cells. Moreover, 5-Aza-treated CD4^+^CD25^h^ T cells showed potent suppressive activity in a cell contact-dependent manner and reduced methylation in the Treg-specific demethylated region (TSDR) in the FOXP3 gene. The analysis of cytokine production revealed that CD4^+^CD25^−^ T cells with 5-Aza treatment produced comparable levels of interferon (IFN)-γ and transforming growth factor (TGF)-β, but less IL-10 and more IL-2, when compared to cells without 5-Aza treatment. The increased IL-2 was indispensible to the enhanced FOXP3 expression in 5-Aza-treated CD4^+^CD25^h^ cells. Finally, 5-Aza-treated CD4^+^CD25^h^ T cells could be expanded with IL-2 supplementation alone and maintained FOXP3 expression and suppressor function through the expansion. Our findings demonstrate that DNA demethylation can enhance the induction of human Treg cells and promise to solve one of the challenges with using Treg cells in therapeutic approaches.

## Introduction

The subset of CD4^+^ T lymphocytes called regulatory T (Treg) cells have been demonstrated to prevent autoimmune disease and transplant rejection in human and experimental animal models (1, 2). Over the last decade, intense study of Treg cell gene expression, surface markers, and suppressor function has revealed two Treg populations. Natural Treg (nTreg) cells are CD4^+^ T cells with strong T cell receptor (TCR) signaling that survive negative selection, develop, and mature in the thymus. Adaptive Treg cells are induced in the periphery following specific tolerogenic stimulation (3). The forkhead winged-helix transcription factor FOXP3 has been shown to be critical for Treg cell differentiation and function. Ectopic expression of FOXP3 confers suppressive function and activates Treg cell signature genes, such as *Il2ra* (CD25), *Ctla4* (CTLA-4), and *Tnfrsf18* (GITR), in peripheral CD4^+^CD25^−^ T cells (4, 5). Activation of human CD4^+^CD25^−^ T cells through TCR stimulation results in transient, low level expression of FOXP3 without conferring suppressive activity (6, 7), indicating that FOXP3 must be constitutively expressed to maintain Treg cell function.

Various protocols have been developed to induce Treg cells from naive CD4^+^CD25^−^ T cells. These include using a variety of APCs such as tolerogenic agent-treated DC (8–10) and plasmacytoid DC (11), cytokines such as transforming growth factor (TGF)-β (12) and IL-35 (13), and suboptimal antigenic activation (14). These efforts have produced FOXP3 expression and suppressor function of variable strength and stability. It has been reported that DNA demethylation in the *foxp3* gene controls FOXP3 expression (15, 16) and the *foxp3* methylation state discriminates bona fide Treg cells from activated FOXP3^+^ CD4^+^ T cells (17). Furthermore, several factors critical for Treg cell development, such as IL-2 receptor alpha chain (also called CD25) (18) and galectin-1 (19), are also regulated by the methylation of CpG islands in the respective promoter regions. These studies indicate that the induction of Treg cells *in vitro* may be enhanced by modifying the ability of CD4^+^ T cells to demethylate DNA.

The typical inhibitor of DNA methyltransferase, 5-azacytidine (5-Aza), is a derivative of the nucleoside cytidine and approved by the FDA to treat myelodisplastic syndrome (MDS) (20). Some studies demonstrated that 5-Aza is capable of inducing strong expression of FOXP3 in mouse CD4^+^CD25^−^ T cells (15, 16, 21, 22). Similar results were also observed in human CD4^+^CD25^−^ T cells (16, 23). However, these proposed 5-Aza-induced FOXP3^+^ T cells has not been fully characterized, and their functionality is controversial. The aim of the present study was to determine if 5-Aza treatment can promote the induction of human CD4^+^CD25^h^FOXP3^+^ T cells from CD4^+^CD25^−^ T cells through suboptimal activation. Here, we show that the FOXP3 and other Treg cell-related markers, as well as the suppressor function of CD4^+^CD25^h^ T cells, were enhanced by 5-Aza treatment, which triggered partial demethylation of Treg-specific demethylated region (TSDR) within the FOXP3 gene. The 5-Aza-treated CD4^+^CD25^h^ T cells were hyporesponsive to TCR engagement and did not produce IL-2 after restimulation. Moreover, 5-Aza-treated induced Treg cells could be expanded with exogenous IL-2 alone and retained FOXP3 expression and their suppressive activity after expansion.

## Materials and Methods

### Blood Samples

Adult peripheral blood obtained from healthy volunteers was acquired in accordance with the approval of Medical Ethics and Human Clinical Trial Committee of the Chung Gung Memorial Hospital. All subjects who were participated in this study gave written informed consent in accordance with the Declaration of Helsinki.

### Isolation of CD4^+^CD25^−^ T Cells

Peripheral blood mononuclear cells (PBMCs) were isolated by density gradient centrifugation over Ficoll-Paque (GE Healthcare) at 3000 rpm for 16 min. CD4^+^CD25^−^ T cells were separated using a magnetic cell sorting (MACS) system (Miltenyi-Biotec). Briefly, CD4^+^ T cells were isolated from PBMCs by negative selection using an LD column. Purified CD4^+^ T cells were subsequently incubated with anti-CD25 antibody-coated beads, and CD4^+^CD25^−^ and CD4^+^CD25^+^ T cell fractions were separated by an MS column. The purity of isolated population was over 95% as determined by FACS analysis.

### Cell Culture

CD4^+^CD25^−^ T cells isolated from PBMC of healthy donors were cultured at 1 × 10^6^ cells/ml in RPMI1640 supplemented with 2 mM l-glutamine, 1% pyruvate, 100 U/ml penicillin, 100 μg/ml streptomycin (Thermo Fisher Scientific), 50 μM 2-mercaptoethanol (Sigma-Aldrich), and 10% heat-inactivated fetal bovine serum (FBS, GE Healthcare). The cells were activated by incubation with anti-CD2, anti-CD3, and anti-CD28 antibodies-coated beads (Miltenyi-Biotec) at a beads-to-cells ratio of 1:8 in the presence or absence of 5 μM 5-Aza (Sigma-Aldrich) for 4 days. For the examination of IL-2 effects on FOXP3 expression, neutralizing anti-human IL-2 and the isotype control antibodies (40 μg/ml) (Biolegend) were added to the cultures with 5-Aza treatment, respectively, at the beginning of the culture. Furthermore, recombinant human IL-2 (100 U/ml) (PeproTech) was also added to 5-Aza-untreated cultures. At the end of culture, cells were harvested for cellular characterization, and supernatants were collected for cytokine measurement.

### Flow Cytometric Analysis

Harvested T cells were washed with 1× PBS (Sigma-Aldrich). The cell suspensions were then stained for 30 min at 4°C with the following mixtures of monoclonal antibodies (mAbs) against human antigens: (i) anti-CD4-FITC (clone PRA-T4), anti-CD25-APC (clone BC96), and anti-GITR-PE (clone 621), anti-CTLA-4-PE (clone BNI3), anti-HLA-DR-PE (clone L243), anti-LAP-PE (clone 27232), or anti-CD127-PE (clone hIL-7R-M21) and (ii) anti-CD4-PE (clone PRA-T4), anti-CD25-APC (Clone BC96), and anti-CD62L-FITC (clone DREG-56) or anti-CD134-FITC (clone ACT35). After staining, cells were washed and resuspended in 1× PBS. For intracellular FOXP3, perforin and granzyme B staining, cells were stained with anti-CD4-FITC and anti-CD25-APC or anti-CD4-FITC and anti-CD25-PE (clone BC96) and were next processed with FOXP3/Transcription Factor Staining Buffer Set (eBioscience) according to the manufacturer’s instructions. Anti-FOXP3-PE (clone PCH101) or anti-perforin-PE (clone δG9) was added into cells stained with anti-CD4-FITC and anti-CD25-APC, and anti-granzyme B-Alexa Fluor 647 (clone GB11) was added into cells stained with anti-CD4-FITC and anti-CD25-PE. Incubation was performed at 4°C for 30 min. For intracellular IL-2 staining, cells were restimulated with 50 ng/ml PMA and 0.5 μg/ml ionomycin (Sigma-Aldrich) in the presence of BD GolgiPlug (BD Biosciences) for 4 h before staining. After restimulation, cells were stained with anti-CD4-FITC and anti-CD25-PE followed by fixation and permeabilization as previous description. The cells were then incubated with anti-IL-2-APC (clone MQ1-17H12) at 4°C for 30 min. Data were acquired on a FACSCalibur flow cytometer (BD Biosciences) and analyzed using the Cell-Quest software (BD Biosciences). Anti-CD4, anti-CD25, anti-CD62L, anti-GITR, anti-HLA-DR, and anti-IL-2 were purchased from Biolegend; anti-CD127, anti-CD134, anti-perforin, and anti-granzyme B were from BD Pharmingen; anti-CTLA-4 was from Serotec; and anti-FOXP3 was from eBioscience.

### Cytokine Measurement

The concentration of IL-2, IL-10, interferon (IFN)-γ, and TGF-β in the culture supernatants were evaluated with ELISA kit (R&D Systems) according to the manufacturer’s instructions.

### *In Vitro* Suppression Assay

Based on the expression intensity of CD25 molecule, CD25^−^, CD25^dim^, and CD25^h^ (the expression intensity of CD25^h^ was threefold higher than that of all CD25^+^ population) were sorted from 5-Aza-treated or 5-Aza-untreated T cells by FACSAria cell-sorting system (BD Biosciences) to be suppressor cells. Freshly isolated CD4^+^ T cells designated as responder cells were stimulated with anti-CD2, anti-CD3, and anti-CD28 antibodies-coated beads at a beads-to-cells ratio of 1:2 in 96-well round-bottom plates (5 × 10^4^ cells/well). The different cell populations of suppressor cells were added to the culture at responders-to-suppressors ratios (R/S) of 1:1, 1:0.5, and 1:0.25 in the final volume of 200 μl complete RPMI for 3 days. The wells were pulsed with 1 μCi of ^3^H-thymidine (PerkinElmer) 16 h before harvesting. The results were measured with Topcount™ Microplate Scintillation and Luminescence Counter (Packard Instrument Company). For transwell assay, the responder cells were labeled with CFSE (5 μM, Thermo Fisher Scientific) for 10 min at 37°C. CFSE-labeled responder cells were stimulated with anti-CD2, anti-CD3, and anti-CD28 antibodies-coated beads at a 1:2 beads-to-cells ratio in 24-well plates (2.5 × 10^5^ cells/well). The suppressor cells were added to the culture at a 1:1 R:S ratio but separated from the responder cells by a Millicell insert (Millipore) for 3 days. The CFSE dilution was analyzed using a FACSCalibur cytometer.

### RNA Isolation and mRNA Expression Analysis

Total RNA was isolated from CD4^+^CD25^−^ and CD4^+^CD25^h^ cells that were sorted from 5-Aza-treated or 5-Aza-untreated T cells using a combination of TRIzol reagent (Thermo Fisher Scientific) and chloroform to purify the RNA. The quantity of RNA was determined by Nanodrop ND-1000 (Thermo Fisher Scientific). For cDNA synthesis, 1 μg of RNA was mixed with 10× reaction buffer and DNase I (Thermo Fisher Scientific) and incubated at 37°C for 30 min. DNase I was inactivated by incubation with 50-mM EDTA at 65°C for 10 min. The DNase I-treated RNA was then mixed with random hexamers (Thermo Fisher Scientific) and incubated at 70°C for 10 min. cDNA was synthesized with M-MLV Reverse Transcriptase (Thermo Fisher Scientific) at 37°C for an hour, and the reaction was inactivated at 70°C for 10 min. Real-time PCR was performed using SYBR Green PCR Master Mix (Thermo Fisher Scientific). RNA expression was measured using the following primers:

FOXP3 forward, 5′-CCCCTGGAGAGCCCAGCCAT-3′
FOXP3 reverse, 5′-GGCACAGCCGAAAGGGTGCT-3′
GAPDH forward, 5′-GAAGGTGAAGGTCGGAGTC-3′
GAPDH reverse, 5′-GAAGATGGTGATGGGATTTC-3′.

### DNA Extraction and DNA Methylation Analysis

DNA was extracted from CD4^+^CD25^h^ cells that were sorted from suboptimally activated T cells treated with or without 5-Aza, and CD4^+^CD25^−^CD127^+^ naive T cells and CD4^+^CD25^h^CD127^−^ nTreg cells that were sorted from freshly isolated PBMCs using a GenElute Mammalian Genomic DNA Miniprep Kit (Sigma-Aldrich) according to the manufacturer’s instructions. For quantification of TSDR methylation, 500 ng of DNA was bisulfite-converted using a EpiTect Fast Bisulfite Conversion Kit (Qiagen) according to the manufacturer’s guidelines. The bisulfite DNA was used for two rounds of PCR using a PyroMark PCR Kit (Qiagen) with the following primers: forward 5′-TTGTTGTAGGATAGGGTAGTTAGT-3′ and reverse Biotin-5′-CAACCCCCCACTTACCCAAATTTTT-3′. PCR was performed with the following parameters: denaturation at 95°C for 15 min, followed by 10 cycles at 95°C for 30 s, 62°C for 30 s, and 72°C for 30 s and another 40 cycles at 95°C for 30 s, 58°C for 30 s, 72°C for 30 s, and the final elongation at 72°C for 7 min. The pyrosequencing procedure was performed on a PyroMark Q24 (Qiagen) according to the manufacturer’s protocol, including the PCR product, PyroMark Gold Q24 reagents, binding buffer (Qiagen), Streptavidin Sepharose beads (GE Healthcare), and the sequencing primer 5′-AGGATAGGGTAGTTAGTT-3′. The methylation rate was determined by the PyroMark Q24 software.

### Cell Lysate Preparation and Immunoblotting

Naive CD4^+^CD25^−^ T cells (1 × 10^6^ cells/ml) with suboptimal TCR stimulation were treated with or without 5-Aza in the 24-well plate. Cells were harvested 4 days later and incubated with lysis buffer containing 0.1M NaCl, 20 mM Tris–HCl, 5 mM MgCl_2_, 0.5% NP-40, and protease inhibitor (Sigma-Aldrich) on ice for 30 min. Cell lysates were collected after centrifugation performed at 16,000 × *g* for 30 min at 4°C. The lysates were separated by 8% SDS-PAGE and transferred to nitrocellulose membranes (GE Healthcare). Antibodies against DNMT1 (Santa Cruz) and β-actin (Sigma-Aldrich) were used to visualize the corresponding proteins.

### Statistical Analysis

Statistical analyses were performed using Prism software (GraphPad). Mann–Whitney *U* test was used to compare all data. These data are presented as means ± SD. *P* values <0.05 were considered statistically significant.

## Results

### Suboptimal TCR Stimulation Combined with 5-Aza Treatment Induced More Human CD4^+^CD25^h^FOXP3^+^ T Cells from CD4^+^CD25^−^ T Cells

**Figure 1:**
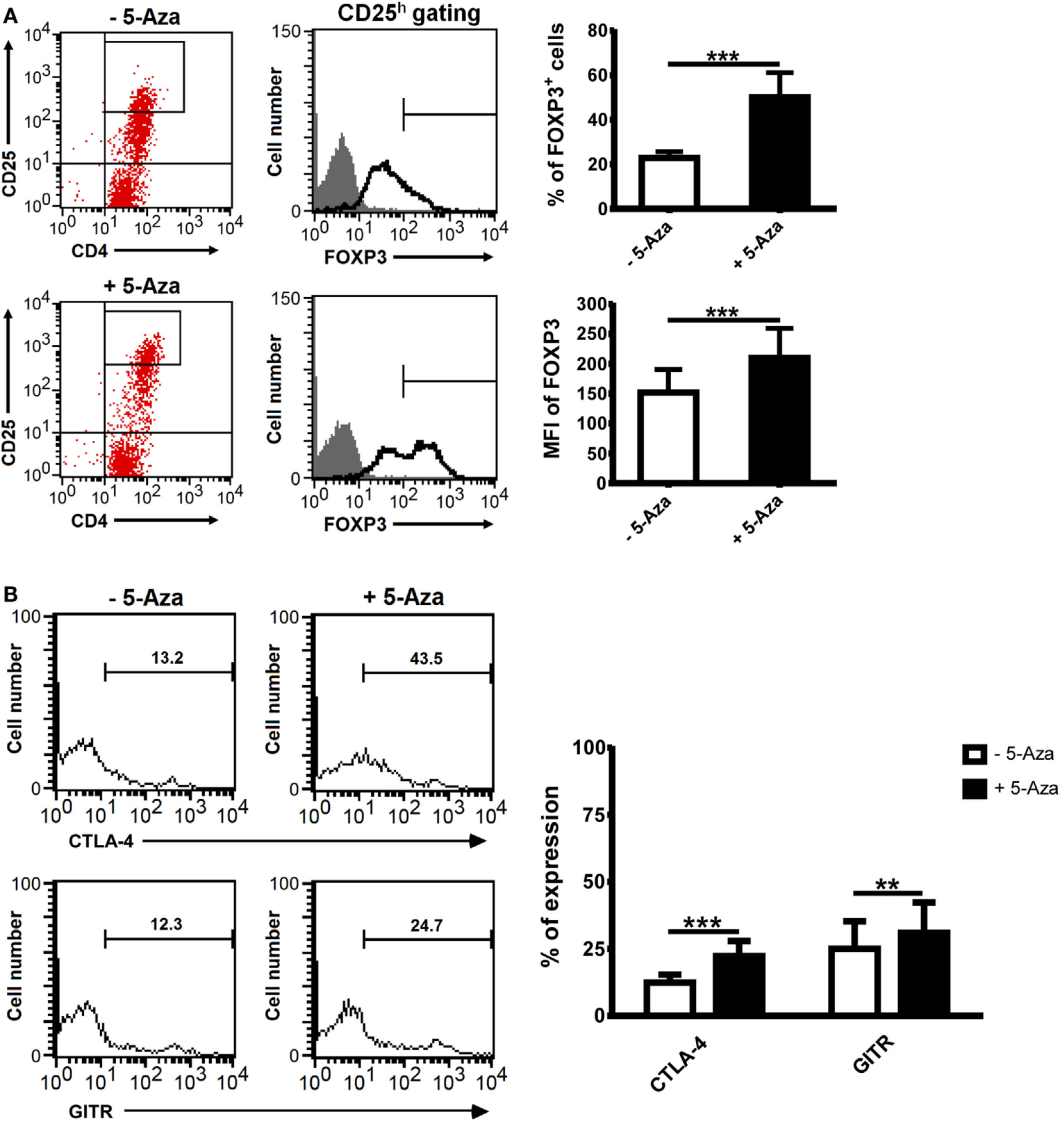
**The treatment of 5-Aza enhanced the expression of Treg-related markers**. CD4^+^CD25^−^ T cells were activated with anti-CD2/CD3/CD28 antibody-coated beads at a bead-to-cell ratio of 1:8 in the presence or absence of 5-Aza for 4 days. **(A,B)** CD25 highly expressing cells were gated to examine the level of FOXP3, CTLA-4, and GITR expression by FACSCalibur. Considering high level of non-specific staining of FOXP3 antibody PCH101, FOXP3^dim^ cells were excluded. One representative sample of nine experiments is shown. The results are shown as mean ± SD (*P < 0.05; **P < 0.01; ***P < 0.001).

To investigate if DNA demethylation can enhance Treg cell induction, CD4^+^CD25^−^ T cells isolated from PBMCs were stimulated with anti-CD2/CD3/CD28-coated beads at different bead-to-cell ratios in the presence or absence of 5-Aza for 4 days. Under suboptimal stimulation, 5-Aza-treated T cells expressed CD25 at much higher levels compared to -untreated ones (Figure S1A in Supplementary Material). To confirm that CD25 expression correlated to Treg cells, we measured the levels of the Treg master transcription factor FOXP3. FOXP3 expression was low in CD25^dim^ cells and barely detectable in CD25^−^ cells with or without 5-Aza treatment (data not shown). The clone of the antibody we used to detect FOXP3 was PCH101, which has been reported to have high level of non-specific staining (24). Thus, we gated FOXP3^h^ and excluded FOXP3^dim^ cells for the analysis. In both treated and untreated CD4^+^CD25^h^ T cells, FOXP3 expression was detected. Interestingly, treatment with 5-Aza increased the frequency of FOXP3^+^ cells, as well as the level of FOXP3 expression per cell (Figure 1A), suggesting inhibition of DNA methyltransferase can enhance CD4^+^CD25^h^FOXP3^+^ T cell induction.

Numerous surface markers have been discovered to be expressed on Treg cells, and many, such as CTLA-4 and GITR, have been implicated as crucial to their function. The percentages of CTLA-4 and GITR expressing cells were significantly increased in 5-Aza-treated CD4^+^CD25^h^ T cells compared to -untreated counterparts (Figure 1B). Other Treg cell-related surface markers, such as CD62L, CD127, CD134, HLA-DR, and LAP, were expressed similarly between 5-Aza-treated and -untreated CD4^+^CD25^h^ cells (Figure S1B in Supplementary Material). These data indicated that 5-Aza enhanced the expression of molecules essential to Treg cell development in human T cells with suboptimal TCR stimulation.

### 5-Aza-Treated CD4^+^CD25^h^ T Cells Suppressed Allogeneic CD4^+^ T Cell Activation

**Figure 2:**
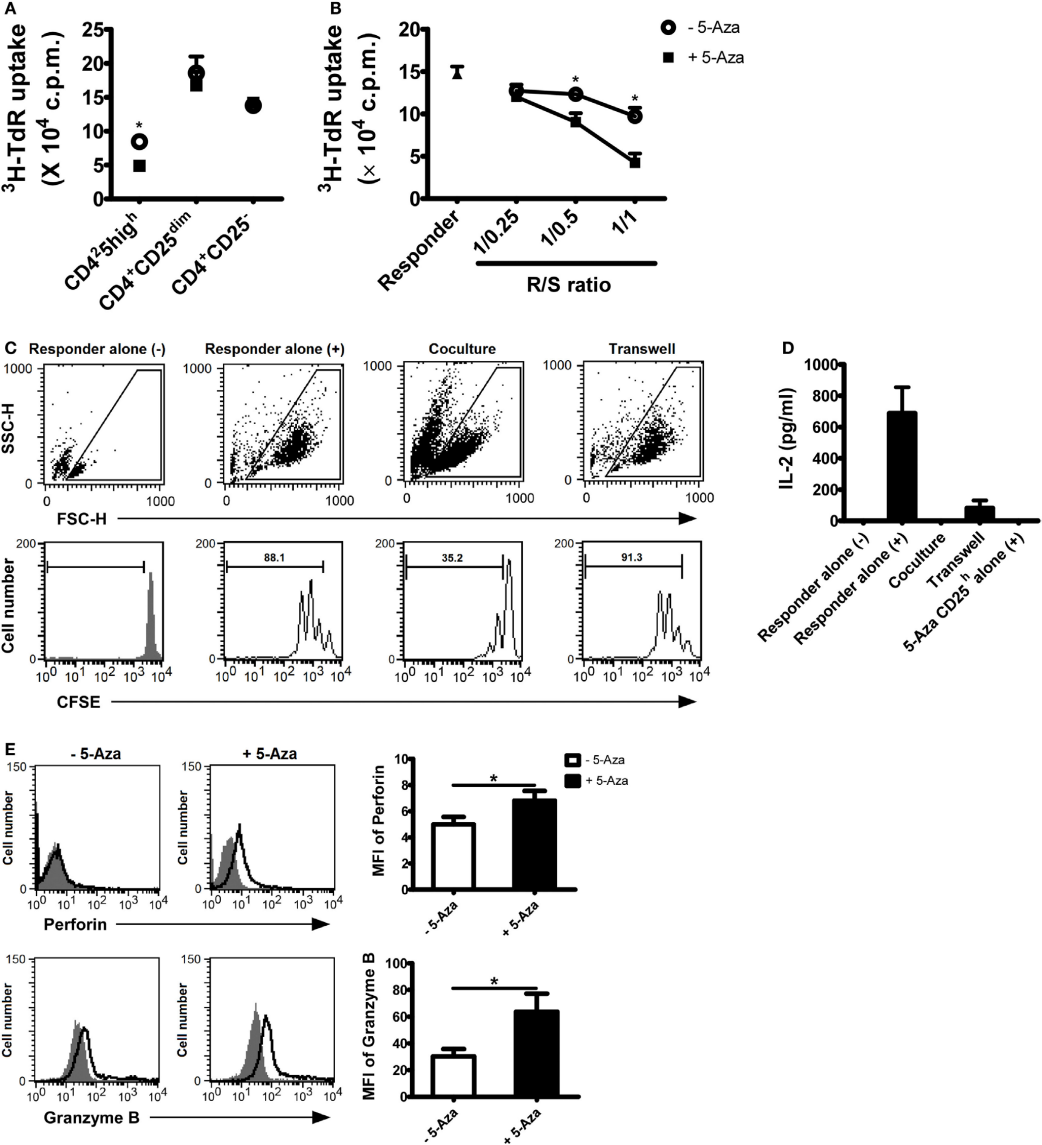
**5-Aza-treated CD4^+^CD25^h^ T cells were hyporesponsiveness to TCR stimulation and demonstrated potent suppressive function**. **(A)** After suboptimal TCR stimulation under the influence of 5-Aza, CD4^+^CD25^h^, CD4^+^CD25^dim^, and CD4^+^CD25^−^ T cells were sorted by FACSAria. The different cell populations were restimulated with antibody-coated beads at a bead-to-cell ratio of 1:2 for 3 days. Proliferation was determined at day 3, with [^3^H]-thymidine added for the last 16 h of culture. **(B)** Allogeneic CD4^+^ T cells (responder) were cocultured with CD4^+^CD25^h^ T cells at the indicated ratios. Proliferation was also determined at day 3 with [^3^H]-thymidine addition. **(C,D)** The responder was incubated with CFSE first and cocultured with 5-Aza-treated CD4^+^CD25^h^ T cells for 3 days. (–) and (+) represent cells without activation and cells stimulated with activation beads, respectively. The results were analyzed by FACSCalibur. Supernatants were collected for IL-2 measurement by ELIA. **(E)** After suboptimal TCR stimulation under the influence of 5-Aza, CD25 highly expressing cells were gated to examine the expression of perforin and granzyme B by FACSCalibur. Data are representative of seven independent experiments and shown as the mean ± SD (*P < 0.05).

Hyporesponsiveness and the ability to suppress CD4^+^ T cells are definitive characteristics of Treg cells. To examine the response of 5-Aza-treated and -untreated CD4^+^CD25^h^ T cells, CD4^+^CD25^h^, CD4^+^CD25^dim^ and CD4^+^CD25^−^ T cells were sorted either from 5-Aza-treated or -untreated T cells and re-activated with optimal TCR stimulation (Figure 2A). As expected, in both 5-Aza-treated and -untreated groups, CD4^+^CD25^dim^ and CD4^+^CD25^−^ T cells were highly proliferative when stimulated, while CD4^+^CD25^h^ T cells showed much more diminished response. Remarkably, 5-Aza-treated CD4^+^CD25^h^ T cells exhibited weaker proliferation than -untreated CD4^+^CD25^h^ T cells, suggesting that DNA methyltransferase inhibition can enhance the characteristic hyporesponsiveness of induced Treg cells.

To further investigate the effects of 5-Aza on the suppressor function of CD4^+^CD25^h^ T cells, freshly isolated allogeneic CD4^+^ T cells, defined as responder cells, were optimally activated for 3 days in the presence of 5-Aza-treated or -untreated CD4^+^CD25^h^ T cells at the indicated ratios. Responder cells were more potently suppressed when cocultured with 5-Aza-treated CD4^+^CD25^h^ than with 5-Aza-untreated CD4^+^CD25^h^ T cells (Figure 2B). Neither CD4^+^CD25^dim^ nor CD4^+^CD25^−^ T cells exhibited any suppressive function themselves, irrespective of 5-Aza exposure (Figures S2A,B in Supplementary Material), strongly indicating that Treg cell induction can be greatly enhanced by inhibiting DNA methylation. Indeed, when comparing the suppressive ability of 5-Aza-treated CD4^+^CD25^h^ T cells with that of nTreg cells, 5-Aza-treated CD4^+^CD25^h^ T cells showed stronger suppression at the R/S of 1/1 despite no statistical significance (Figure S3 in Supplementary Material).

It has been demonstrated that Treg cells exert their regulatory function through cell-to-cell contact *in vitro*. We next used transwell assays to verify the inhibitory mechanism of 5-Aza-treated CD4^+^CD25^h^ T cell. CFSE-labeled responder cells expanded largely upon optimal TCR stimulation (Figure 2C). As expected, proliferation was dramatically hindered when responder cells were cocultured with 5-Aza-treated CD4^+^CD25^h^ Treg cells. However, the suppression was abolished when the two cell populations were separated by a semi-permeable insert. Similarly, 5-Aza-treated CD4^+^CD25^h^ Treg cells were able to suppress responder cell production of IL-2, although IL-2 production did not recover completely when cell-to-cell contact was prevented (Figure 2D). After stimulation, 5-Aza-treated CD4^+^CD25^h^ T cells, which we had previously demonstrated their hyporesponsive characteristic, did not produce IL-2 (Figure 2D). Further examination of the molecules that have been proposed to determine Treg cell functionality revealed that perforin and granzyme B were dramatically upregulated in 5-Aza-treated CD4^+^CD25^h^ Treg cells (Figure 2E). These data demonstrated the potential application of 5-Aza to induce human CD4^+^CD25^−^ T cells into a population of CD4^+^CD25^h^ T cells capable of regulating immune responses.

### Partial Demethylation in the TSDR with Increased *foxp3* Gene Expression and Decreased Level of DNMT1 Were Observed in 5-Aza-Treated CD4^+^CD25^h^ T Cells

**Figure 3:**
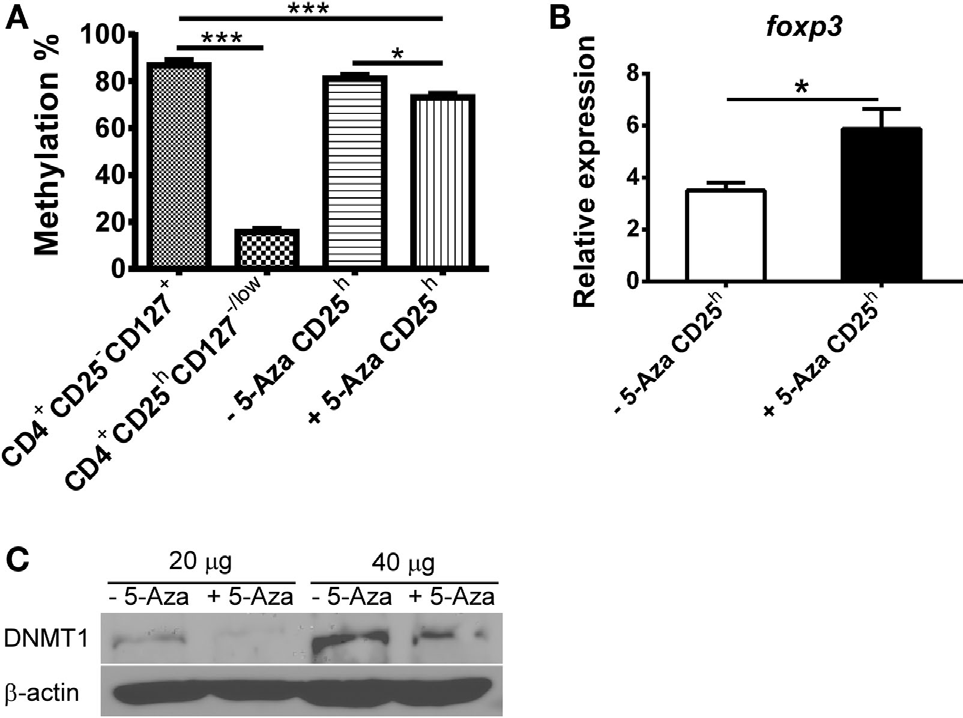
**Treg-specific demethylating region in 5-Aza-treated CD4^+^CD25^h^ T cells is partially demethylated in company with higher *foxp3* expression due to decreased DNMT1**. **(A)** CD4^+^CD25^h^ T cells were sorted from 5-Aza-treated and -untreated cells. Other cell populations were CD4^+^CD25^−^CD127^+^ and CD4^+^CD25^h^CD127^−/low^ T cells sorted from CD4^+^ T cells which were separated from freshly isolated PBMCs. Genomic DNA was extracted, and the TSDR methylation pattern was analyzed by pyrosequencing. **(B)** RNA was extracted from CD4^+^CD25^−^ and CD4^+^CD25^h^ T cells, which were sorted from 5-Aza-treated and -untreated cells for detection of *foxp3*, and *GAPDH* expression by real-time PCR. Data are representative of five independent experiments and shown as the mean ± SD (*P < 0.05; **P < 0.01; ***P < 0.001). **(C)** Total proteins were extracted from 5-Aza-treated and -untreated cells for detection of DNMT1 and β-actin by western blot. One representative result of three experiments is shown.

The TSDR in the *foxp3* loci has been proposed to be hypomethylated in Treg cells. Demethylation within TSDR is not seen in activated T cells that transiently express FOXP3 (17, 25). To elucidate the effect of the methyltransferase inhibitor 5-Aza on Treg induction, we examined the *foxp3* methylation pattern in T cells. Consistent with previous reports, we found that the TSDR was hypomethylated in nTreg but hypermethylated in naive T cells (Figure 3A). Partial demethylation was observed in 5-Aza-treated CD4^+^CD25^h^ cells, but the hypermethylated pattern was not changed in 5-Aza-untreated CD4^+^CD25^h^ cells. To investigate the effect of demethylation by 5-Aza treatment on gene expression, we analyzed total RNA extracted from 5-Aza-treated and -untreated CD4^+^CD25^h^ T cells. As shown in Figure 3B, *foxp3* was upregulated in 5-Aza-treated CD4^+^CD25^h^ T cells compared to the 5-Aza-untreated cells. The expression of *dnmt1, dnmt3a*, and *dnmt3b* was also examined. They were expressed comparably between the 5-Aza-treated and -untreated cell populations (data not shown). Although the expression of *dnmt* family genes was not affected, we found that protein amounts of DNMT1 were downregulated in 5-Aza-treated T cells, but not in their counterparts (Figure 3C). These data showed that decrease of DNA methylation in naive T cells through reduction of DNMT1 by 5-Aza increased FOXP3 expression and potentiated their regulatory function.

### IL-2 Was Indispensible to the Enhanced FOXP3 Expression in 5-Aza-Treated CD4^+^CD25^h^ T Cells

**Figure 4:**
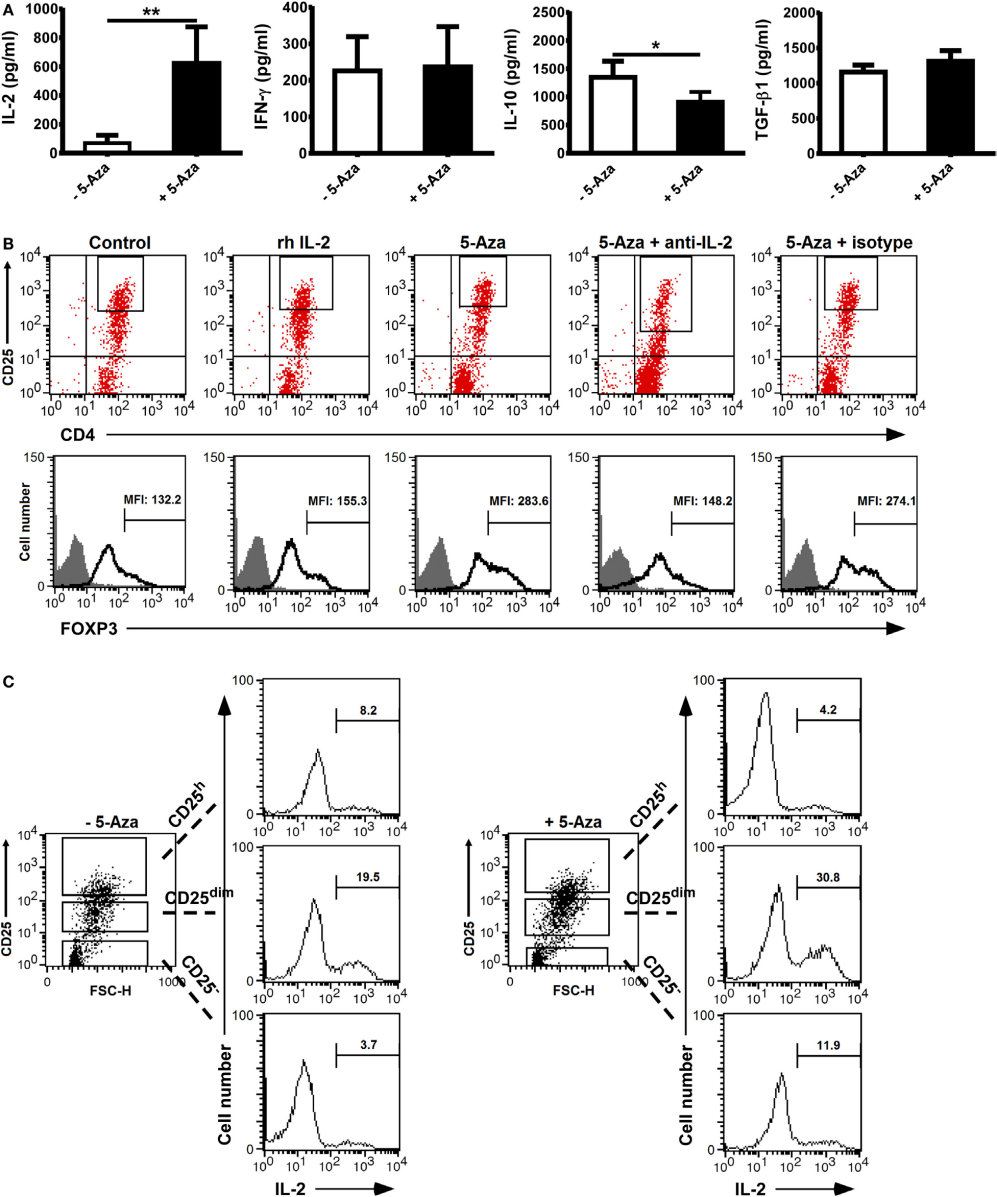
**Neutralization of IL-2 which was majorly produced by CD25^dim^ cells impeded the enhanced FOXP3 expression in CD25^h^ cells treated with 5-Aza**. CD4^+^CD25^−^ T cells were activated with anti-CD2/CD3/CD28 antibody-coated beads at a bead-to-cell ratio of 1:8 in the presence or absence of 5-Aza for 4 days. **(A)** IL-2, IFN-γ, IL-10, and TGF-β in the cultured supernatants were measured by ELISA. Data are representative of nine independent experiments. The results are shown as mean ± SD (*P < 0.05; **P < 0.01). **(B)** Neutralizing anti-IL-2 antibodies and recombinant IL-2 were added simultaneously to the cells treated with 5-Aza and without 5-Aza, respectively. FOXP3 was examined in the CD25^h^ cells by FACSCalibur, and FOXP3^dim^ cells were excluded. **(C)** After the incubation with 5-Aza, cells were restimulated with PMA and ionomycin for an hour followed by GolgiPlug treatment for another 3 h. Intracellular IL-2 was analyzed by FACSCalibur. One representative result of six **(B)** and three **(C)** experiments is shown.

Cytokines, such as IL-2 or TGF-β, have important effects on the development and function of Treg cells. We hence studied the cytokine production of suboptimally activated T cells under the influence of 5-Aza. As shown in Figure 4A, the level of IL-2 produced by 5-Aza-treated, suboptimally activated T cells was higher, but the level of IFN-γ was similar, compared to -untreated T cells. Next, we analyzed the levels of the inhibitory cytokines IL-10 and TGF-β. The production of IL-10 was reduced when T cells were treated with 5-Aza, whereas that of TGF-β was comparable (Figure 4A). Since IL-2 is critical to the development and maintenance of Treg cells, we tested to see if the increased IL-2 production from 5-Aza-treated cells might be important for their enhanced FOXP3 expression. We utilized anti-IL-2 antibody to neutralize IL-2 production in the culture with 5-Aza treatment. In addition, we examined whether exogenous IL-2 can augment FOXP3 expression by adding recombinant IL-2 to the culture without 5-Aza influence. As shown in Figure 4B, IL-2 blockade eliminated the enhanced FOPX3 expression previously observed in 5-Aza-treated CD4^+^CD25^h^ T cells. However, exogenously supplemented IL-2 did not augment FOXP3 expression in suboptimally activated, 5-Aza-untreated CD4^+^CD25^h^ T cells, which altogether suggested that IL-2 production was indispensable to the enhanced Treg induction effect of DNA demethylation. To further investigate the origin of IL-2 secretion, cultured cells were restimulated with PMA and ionomycin in the presence of GolgiStop. After restimulation, IL-2 was barely detectable in CD25^h^ T cells irrespective of 5-Aza treatment. Surprisingly, the major population producing IL-2 was CD25^dim^ T cells, which actually produced more IL-2 when they had been treated with 5-Aza (Figure 4C). These results demonstrated that 5-Aza treatment enhanced the induction of Treg cells from CD4^+^CD25^−^ T cells directly by modifying FOXP3 protein expression in CD25^h^ T cells as well as indirectly by increasing IL-2 production in CD25^dim^ cells.

### 5-Aza-Treated CD4^+^CD25^h^ T Cells Maintained FOXP3 Expression and Their Suppressive Function after 2-Week Expansion with Recombinant IL-2 Supplement

**Figure 5:**
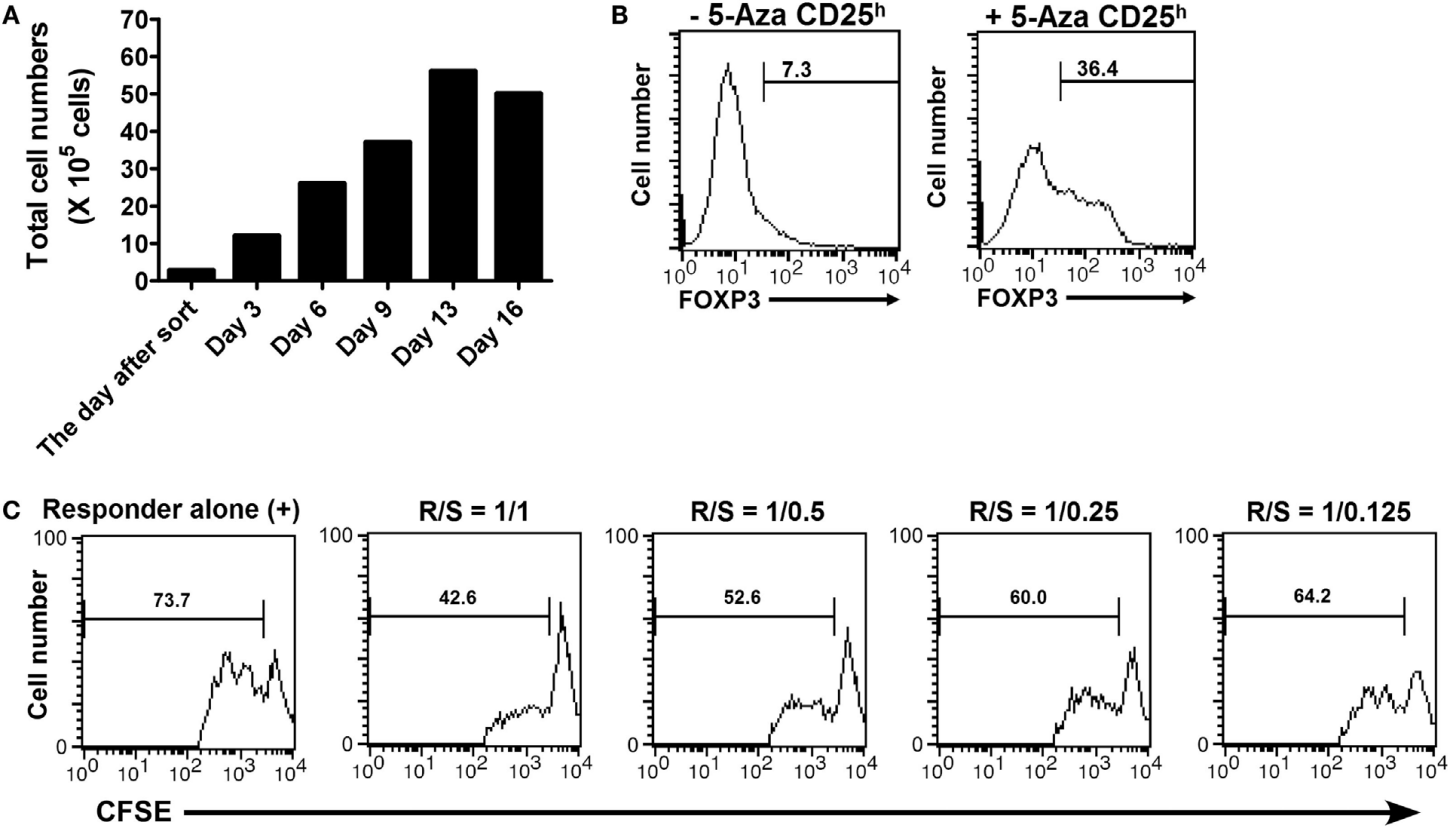
**5-Aza-treated CD4^+^CD25^h^ T cells proliferated without TCR stimulation, but with only IL-2 supplementation and still maintained FOXP3 expression and their suppressive function after expansion**. The sorted 5-Aza-treated CD4^+^CD25^h^ T cells were cultured with recombinant IL-2 (100 U/ml). The culture medium supplied with IL-2 was refreshed every 2 days for 16 days. **(A)** The cell numbers were determined by trypan blue exclusion during the refreshment of the culture medium. **(B)** FOXP3 expression in the expanded CD4^+^CD25^h^ T cells was examined by intracellular staining followed by flow cytometric analysis. FOXP3^dim^ cells were excluded. **(C)** The expanded 5-Aza-treated CD4^+^CD25^h^ T cells were cocultured with CFSE-labeled responder for 3 days. The results were analyzed by FACSCalibur. One representative result of five experiments is shown.

The challenge to inducing Treg cells from human CD4^+^ T cells has been considered how to produce FOXP3^+^ T cells that maintain adequate suppressive function. To assess the ability of 5-Aza treatment to induce Treg cells, we examined FOXP3 expression and the suppressor function of 5-Aza-treated CD4^+^CD25^h^ T cells after *ex vivo* expansion. Sorted 5-Aza-treated CD4^+^CD25^h^ T cells were maintained in culture medium with 100 U/ml of recombinant IL-2 for 16 days. The medium was refreshed and supplemented with recombinant IL-2 (100 U/ml) every 2 days. The cell number was determined by trypan blue exclusion during the every refreshment. 5-Aza-treated CD4^+^CD25^h^ T cells expanded even if provided only with recombinant IL-2, as shown in the Figure 5A. Examination of FOXP3 expression indicated that 5-Aza-untreated CD4^+^CD25^h^ T cells lost FOXP3 expression. Importantly, 5-Aza-treated CD4^+^CD25^h^ T cells still retained FOXP3 expression (Figure 5B). Furthermore, expanded 5-Aza-treated CD4^+^CD25^h^ T cells were able to control the proliferation of responder cells in a dose-dependent manner (Figure 5C). These results indicated that 5-Aza-treated CD4^+^CD25^h^ T cells retained FOXP3 expression and their ability to suppress after expansion with recombinant IL-2 alone.

## Discussion

*Ex vivo* generation of Treg cells promises to revolutionize therapy for promoting tolerance post-transplantation and modulating the severity of autoimmunity and allergy. Heretofore, attempts to induce Treg cells from human CD4^+^ T cells have yet to produce stable FOXP3^+^ T cells capable of maintaining suppression. Activation thresholds have been proposed to be responsible for the peripheral tolerance (26), and suboptimal T-cell activation was found to promote induction of Treg cells in an autonomous TGF-β-dependent manner in murine systems (14, 27). In this study, we have demonstrated that DNA demethylation by 5-Aza can promote Treg cell induction by suboptimal TCR stimulation of human CD4^+^CD25^−^ T cells through increasing IL-2 production in CD25^dim^ and CD25^−^ cells. The increased IL-2 synergistically enhanced FOXP3 expression in CD25^h^ cells. Induced FOXP3^+^ T cells that had been treated with 5-Aza showed all the hallmarks of Treg cells, including expression of CTLA-4 and GITR, downregulated expression of CD127, production of TGF-β, and the capability of suppressing responder T-cells in a contact-dependent manner. Importantly, 5-Aza-treated Treg cells could be expanded *ex vivo* with IL-2 alone and maintained FOXP3 expression and their suppressive function after expansion.

Using DNA methyltransferase inhibitor to induce FOXP3 expression has been extensively studied in mice (16, 22, 28). However, induction of FOXP3 expression with epigenetic modification in human is still controversial. Lal and colleagues reported that CD4^+^CD25^+^FOXP3^+^ T cells were induced from human CD4^+^CD25^−^ T cells by the combination of 5-Aza-2′-deoxycytidine (5-Aza-dC) and TGF-β in the presence of exogenous IL-2, and they performed steady suppressive activity (16). Nevertheless, the resulting cells’ phenotype and regulatory function were not fully characterized. Furthermore, TGF-β-induced FOXP3^+^ cells in human system are still controversial (24, 29). We here show that the combination of suboptimal TCR stimulation and 5-Aza treatment induced a population of human CD4^+^ T cells that expressed higher level of CD25 and FOXP3 as well as CTLA-4 and GITR when compared to the 5-Aza-untreated counterparts, and the amounts of TGF-β in the supernatants of 5-Aza-treated and -untreated groups were comparable, which might suggest the enhanced expression of CD25, FOXP3, CTLA-4, and GITR was TGF-β independent. Despite no significant difference compared to non-5-Aza-treated cells, downregulated expression of CD127 in 5-Aza-treated CD4^+^CD25^h^ T cells corresponded to the level in nTreg cells. Moreover, sorted 5-Aza-treated CD4^+^CD25^h^ T cells not only were hyporesponsive to TCR engagement but also demonstrated potent suppressive activity in a contact-dependent manner, which are the two characteristics of nTreg cells observed *in vitro* (30). It is worth mentioning that the strength of suppression mediated by 5-Aza-treated CD4^+^CD25^h^ T cells *in vitro* is comparable to that of nTreg cells. Still, before 5-Aza-treated CD4^+^CD25^h^ T cells can be called “true” induced Treg cells, their transcription profile has to be compared to that of nTreg cells, and their suppressor function needs to be tested *in vivo* in an immunological setting such as the xenogeneic graft-versus-host disease (GvHD) model in NOD-*scid*-γc^−^ (NSG) mouse (31).

Demethylation of the CpG island in the Treg-specific demethylation region (TSDR) has been reported to promote stable and constitutive FOXP3 expression in nTreg cells (32, 33). However, whether the TSDR is demethylated in the *in vivo* generated peripheral Treg (21, 34) and *in vitro* TGF-β-induced Treg (35–37) remains controversial. Despite only partial demethylation of the TSDR in 5-Aza-treated CD4^+^CD25^h^ cells (but significantly reduced methylation), the level of *foxp3* mRNA expression was increased when compared to the CD4^+^CD25^h^ cells without 5-Aza treatment. In addition, the protein level of DNMT1 in 5-Aza-treated cells was decreased, in agreement with the fact that when 5-Aza incorporated into DNA forms covalent complexes with DNMTs, it results in the degradation of the enzymes (38). Recently, DNA demethylation enzymes, the Tet family members, have been proposed to be involved in regulating FOXP3 expression (39). We have examined the expression of Tet family members in 5-Aza-treated and -untreated CD4^+^CD25^h^ T cells by real-time PCR. The expression of Tet1, Tet2, and Tet3 was decreased in 5-Aza-treated CD4^+^CD25^h^ T cells (data not shown), indicating that Tet proteins might not involve in the enhanced FOXP3 expression in cells treated with 5-Aza.

A previous study reported by Kehrmann et al. indicated that 5-Aza-dC reduced methylation within TSDR and increased FOXP3 expression in the 5-Aza-dC-treated CD4^+^CD25^−^ cells, as well as the mRNA expression of T helper (Th)1-, Th2-, and Th17-related transcription factors (40). They found that the 5-Aza-dC-treated cells did not suppress the proliferation of responder cells, suggesting the loss of suppressive ability of 5-Aza-dC-treated cells despite the increased FOXP3 expression. In our opinion, the culture of CD4^+^CD25^−^ T cells results in a heterogeneous cell population with varying CD25 expression. Isolation of CD25^h^FOXP3^+^ cells is necessary to study the role of 5-Aza-dC treatment on Treg suppressive function. Furthermore, optimal TCR stimulation may trigger the differentiation of naive T cells into effector Th cell populations (41). Contrary to their study, we have demonstrated that sorted 5-Aza-treated CD4^+^CD25^h^ T cells expressed important Treg cell markers, were not responsive to TCR stimulation, and showed potent suppressive function. To address the concern that adult peripheral blood contains a large fraction of effector/memory CD4^+^ T cells, which are CD45RA^−^CD45RO^+^, the addition of CD45RA as a marker to select for pure naive CD4^+^ T cells could significantly raise the efficiency of Treg cell induction.

IL-2 signaling is critically required for the development and homeostasis of Treg cells for dominant immunotolerance *in vivo* (42). Previous studies also emphasized the important role of IL-2 in the conversion of Treg cells from naive cells by TGF-β (37, 43). Our results demonstrated that IL-2 was increased in the 5-Aza-conditioned culture. The IL-2 produced mainly by the CD25^dim^ cells is indispensible to the enhanced expression of FOXP3 in CD25^h^ cells. When blocking IL-2 by neutralizing antibodies, the expression of FOXP3 in 5-Aza-treated CD25^h^ cells decreased to the FOXP3 level expressed by 5-Aza-untreated CD25^h^ cells. Meanwhile, the addition of exogenous IL-2 to cells without 5-Aza treatment did not reinforce FOXP3 expression in CD25^h^ cells, suggesting only suboptimal TCR stimulation is not sufficient to induce competent Treg cells. How the epigenetic changes provoked by 5-Aza requires IL-2 signaling in order to enhance FOXP3 expression in cells remains to be investigated.

To address concerns about the stability of Treg cell function which has been tightly correlated with the methylation status of *foxp3* TSDR (3), we investigated whether 5-Aza-treated CD4^+^CD25^h^ T cells could keep their suppressive ability. Sorted 5-Aza-treated CD4^+^CD25^h^ T cells were maintained in the culture medium supplemented with recombinant human IL-2 for more than 2 weeks. IL-2 alone expanded the 5-Aza-treated CD4^+^CD25^h^ T cells but for up to 20-folds. In addition, the 5-Aza-treated CD4^+^CD25^h^ T cells still maintained FOXP3 expression and their suppressive ability after expansion. Taken together, we have demonstrated a simple and fast approach to induce human Treg cells *in vitro* from CD4^+^CD25^−^ T cells through the combination of suboptimal TCR stimulation and 5-Aza treatment. 5-Aza-treated induced human Treg cells may be used as adoptive immunotherapy to alleviate the severity of diseases caused by unwanted immune reactions, such as allergy, autoimmune disease, transplant rejection, and GvHD.

## Author Contributions

C-HL, C-JW, S-JL, JY, and M-LK designed the research. C-HL performed all experiments, analyzed the data, and prepared the figures. C-CC and K-RL helped with cell sorting. L-CC contributed analytic tools. C-HL, C-JW, DN, and M-LK wrote the paper.

## Conflict of Interest Statement

The authors declare that the research was conducted in the absence of any commercial or financial relationships that could be construed as a potential conflict of interest.
