# Supplementary material for: DNA Methyltransferase Inhibitor Promotes Human CD4+CD25hFOXP3+ Regulatory T Lymphocyte Induction under Suboptimal TCR Stimulation
Source: Front Immunol. 2016 Nov 8;7:488. doi: 10.3389/fimmu.2016.00488 (PMC5099256; doi:10.3389/fimmu.2016.00488)
Supplement: Supplementary file 1 [file Data_Sheet_1.DOCX]

***Supplementary Material***

**DNA Methyltransferase Inhibitor Promotes Human CD4^+^CD25^h^FOXP3^+^ Regulatory T Lymphocyte Induction under Suboptimal TCR Stimulation**

**Chun-Hao Lu, Cheng-Jang Wu, Cheng-Chi Chan, Duc T. Nguyen, Kuo-Ray Lin, Syh-Jae Lin, Li-Chen Chen, Jeffrey Jong-Yong Yen, Ming-Ling Kuo***

*** Correspondence:** Ming-Ling Kuo: [mingling@mail.cgu.edu.tw](mailto:mingling@mail.cgu.edu.tw)

**Supplementary Figures**

**
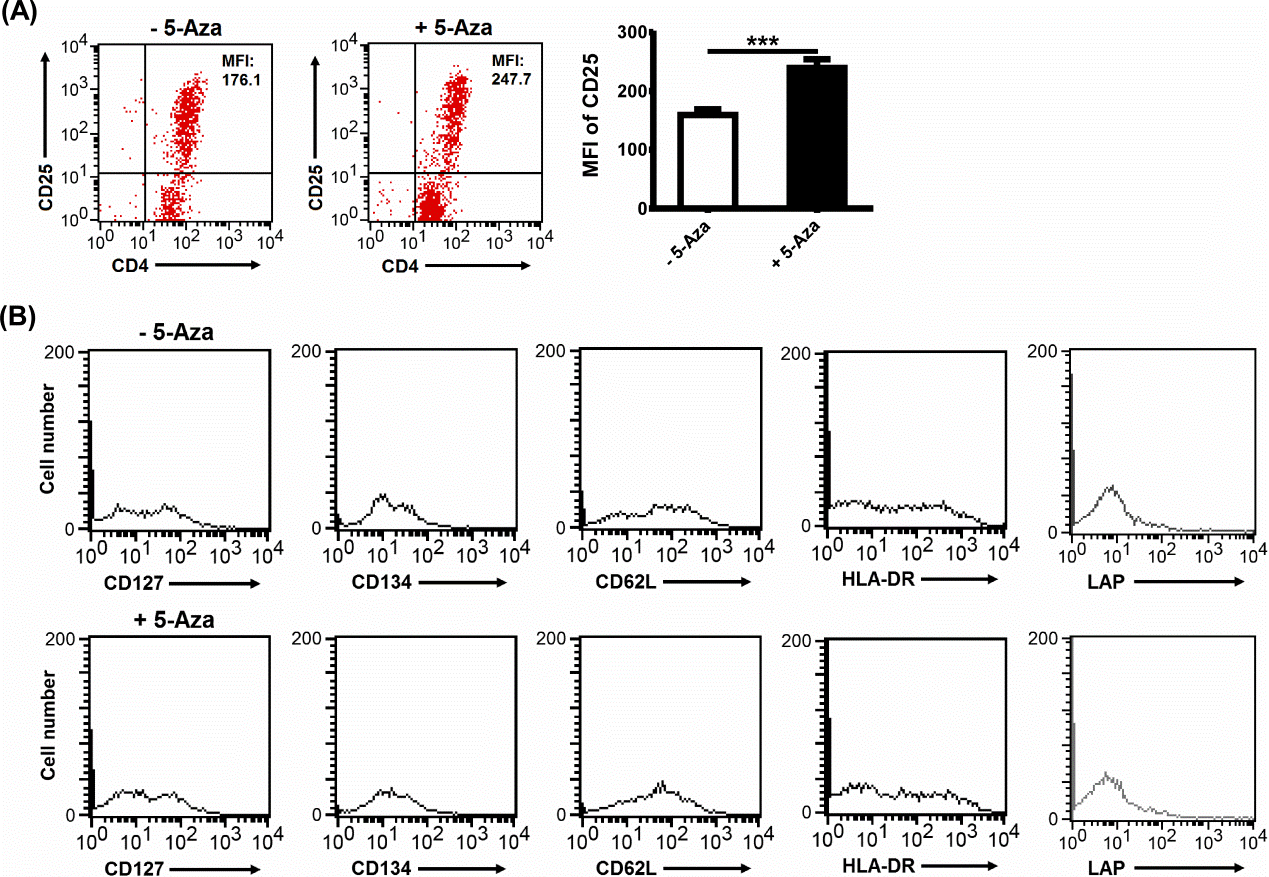
**

**Supplementary Figure 1.** CD25 expression was increased by 5-Aza treatment but not other Treg-related surface molecules. CD4^+^CD25^-^ T cells were activated with anti-CD2/CD3/CD28 antibody-coated beads at a bead-to-cell ratio of 1 to 8 in the presence or absence of 5-Aza for 4 days. (A) The cultured cells were stained with anti-CD4 and anti-CD25 antibodies and analyzed on a FACSCalibur. One representative sample of nine experiments is shown. (B) The indicated Treg-related markers were examined in CD25^h^ cells using FACSCalibur. One representative sample of three experiments is shown. *** *p* < 0.001.

**
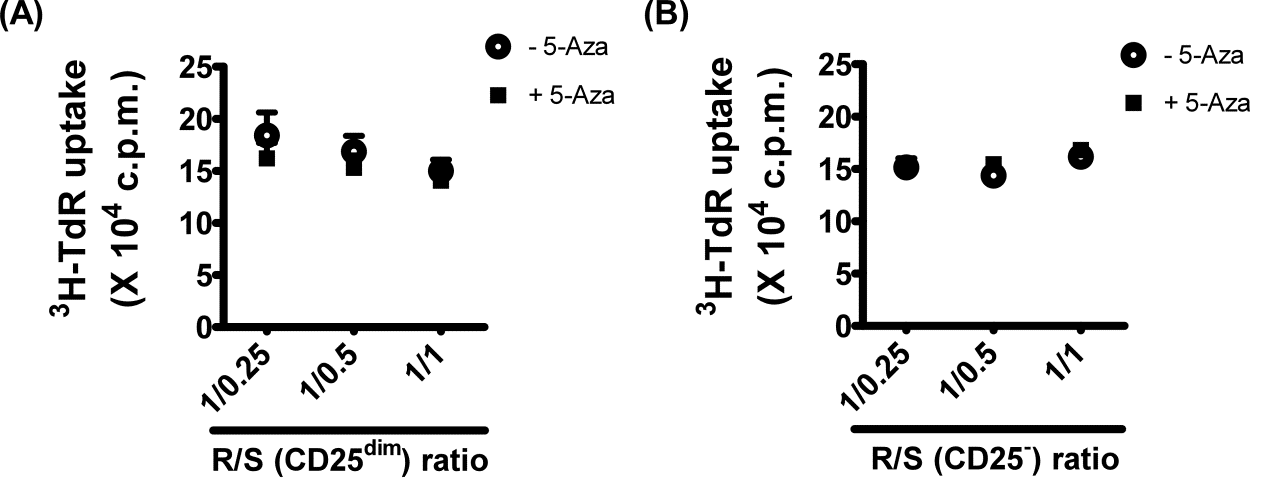
**

**Supplementary Figure 2.** CD4^+^CD25^dim^ and CD4^+^CD25^-^ T cells sorted from cells treated with or without 5-Aza did not have suppressive function. CD4^+^CD25^dim^ (A) and CD4^+^CD25^-^ (B) T cells were sorted from the culture under the influence of 5-Aza by FACSAria. Rresponder cells were cocultured with CD4^+^CD25^dim^ or CD4^+^CD25^-^ T cells at the indicated ratios. Proliferation was determined at day 3 with [^3^H]-thymidine addition for the last 16 h of culture. Data are representative of 7 independent experiments and shown as the mean ± SD.

**
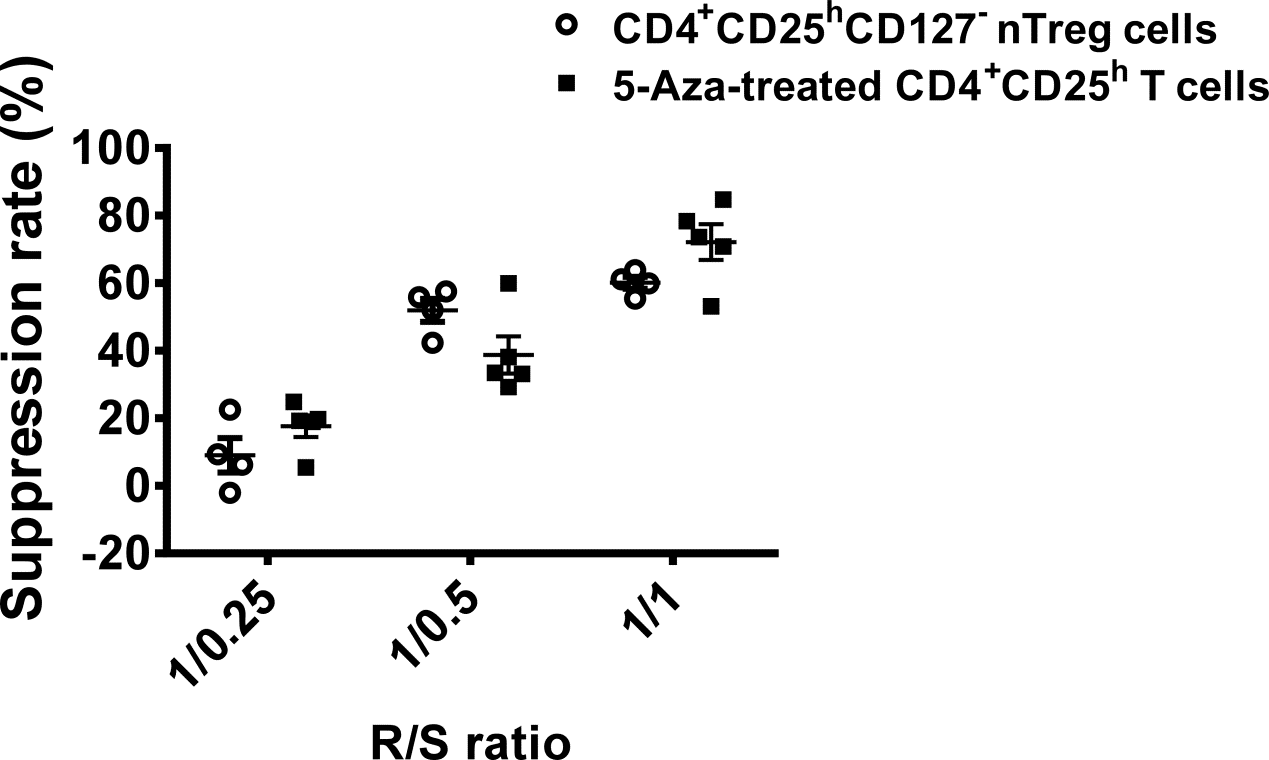
**

**Supplementary Figure 3.** 5-Aza-treated CD4^+^CD25^h^ T cells performed potent suppressive ability comparable to CD4^+^CD25^h^CD127^-^ nTreg cells. CD4^+^CD25^h^CD127^-^ nTreg cells and 5-Aza-treated CD4^+^CD25^h^ T cells were sorted from isolated CD4^+^T cells and suboptimally activated naive T cells in the presence of 5-Aza, respectively. Rresponder cells were cocultured with CD4^+^CD25^h^CD127^-^ or 5-Aza-treated CD4^+^CD25^h^ T cells at the indicated ratios. Proliferation was determined at day 3 with [^3^H]-thymidine addition for the last 16 h of culture. Data are representative of 5 independent experiments and shown as the mean ± SD.
